# Networks of Depression and Anxiety Symptoms Across Development

**DOI:** 10.1016/j.jaac.2018.05.027

**Published:** 2018-12

**Authors:** Eoin McElroy, Pasco Fearon, Jay Belsky, Peter Fonagy, Praveetha Patalay

**Affiliations:** a University of Liverpool, UK; b University College London, UK; c University of California, Davis

**Keywords:** depression, anxiety, comorbidity, developmental psychopathology, transdiagnostic

## Abstract

**Objective:**

Frequent co-occurrence and bidirectional longitudinal associations have led some researchers to question the boundaries between depression and anxiety. A longitudinal investigation of the interconnected symptom structure of these constructs may help determine the extent to which they are distinct, and whether this changes over development. Therefore, the present study used network analysis to examine these symptom−symptom associations developmentally from early childhood to mid-adolescence.

**Method:**

We analyzed data from the National Institute of Child Health and Human Development Study of Early Child Care and Youth Development (N = 1,147). Depression and anxiety symptoms were assessed on 7 occasions between ages 5 and 14 years using maternal reports. Regularized partial correlation networks were constructed at each time point, and diagnostic boundaries were explored using empirical tests of network modularity (ie, clustering of symptom nodes). Nonparametric permutation tests were used to determine whether symptoms became more associated over development, and network centrality was examined to identify developmental changes in the overall importance of specific symptoms.

**Results:**

Symptoms formed highly interconnected networks, as evidenced by strong associations between depression and anxiety symptoms and a lack of distinct clustering. There was some evidence of an increase in overall connectivity as children aged. Feeling “anxious/fearful” and “unhappy/sad” were consistently the most central symptoms over development.

**Conclusion:**

Minimal clustering of nodes indicated no separation of depression and anxiety symptoms from early childhood through mid-adolescence. An increase in connectivity over development suggests that symptoms may reinforce each other, potentially contributing to the high levels of lifetime continuity of these disorders.

Anxiety and depression are the most prevalent forms of psychopathology and contribute substantially to the global burden of disease.^1^, ^2^ These disorders frequently co-occur throughout childhood and adolescence, with comorbidity estimates ranging from 15% to 75%.^3^ Although both disorders have been shown to predict each other over time,^3^ the expression of comorbidity may vary developmentally, given that anxiety is typically more common in childhood, whereas depression is more prevalent in adolescence.^4^ This developmental overlap has led researchers to question whether anxiety and depression should be considered a unitary construct, rather than distinct entities.^5^ Indeed it has been suggested that depression and anxiety may reflect a single construct in early childhood that becomes increasingly differentiated as children age.^5^ This idea is reflected in our most common approaches to measurement, with broader syndromes (internalizing) typically favored in child/adolescent research,^6^ compared to research in adults, which is more disorder focused.^7^ However, the empirical support for this increased differentiation is mixed. For instance, a number of factor-analytic studies have reported the superior fit of unidimensional models in younger children, and separate depression and anxiety factors in older children.^8^, ^9^ Conversely, other studies have found that depression and anxiety could be differentiated across childhood and adolescence.^10^, ^11^

One reason for such inconsistent findings may relate to the manner in which these constructs have been conceptualized and measured. The research to date has used aggregate scores wherein symptoms have been treated as interchangeable indicators of underlying disorders/syndromes.^8^, ^10^ By contrast, relatively little is known about how individual symptoms themselves are related over development. Symptoms within diagnostic constructs are highly heterogeneous, and combining them to form aggregate scores may result in a considerable loss of information.^12^ Indeed, this was evidenced in a study by Boylan *et al.*,^11^ in which they sought to test the factorial invariance of depression and anxiety across childhood using data from a large community sample (N = 1,329). Although a two-factor model fit well across development, a number of individual items failed to show invariance over time, which suggests that the importance of individual items may change as children age.^11^ A better understanding of the interconnected symptom structure of depression and anxiety may help determine the extent to which these constructs are distinct, and whether they become more or less related over development.

Network analysis may be a useful technique in this regard, as it models complex networks of locally associated symptoms.^13^ This approach graphically depicts psychiatric symptoms as *nodes* (ie, points in space)*,* and the estimated associations between symptoms as *edges* (ie, lines denoting strength of effect)*.* Key nodes (ie, those with the most and strongest edges) are placed centrally within networks, meaning that their effects spread quickly throughout the network when activated; less influential nodes are consigned to the periphery.^13^ The main advantage of the network approach is that it allows us to quantify the overall importance of symptoms/nodes (ie, centrality in the network), while also highlighting where symptoms are important (ie, the individual edges, relative positioning in the network). Thus, this approach offers an intuitive explanation for comorbidity in the form of highly associated symptom pairs (referred to as “bridge symptoms”) that serve to link clusters of symptoms^14^ possibly reflecting causal processes linking symptoms, shared etiological influences or a combination of both.^14^, ^15^, ^16^ As such, network analysis may afford insight into the complex associations that exist both within and between constructs, and thereby may be a useful tool in our attempts to illuminate the mechanisms underlying comorbid depression and anxiety.

To our knowledge, only three investigations, all in adult samples, have used network techniques to examine the associations between depression and anxiety symptoms, with inconsistent findings. Beard *et al.*^17^ examined the network structure of depression and anxiety in a sample of adults undergoing treatment (N = 1,029, mean age = 35 years), discerning a high degree of clustering, with distinct depression and anxiety regions bridged by only three edges; “Motor Retardation – Restless,” “Nervous – Sad mood,” and “Guilt – Worry”.^17^ A second study illuminated the network structure of major depression, generalized anxiety disorder, and somatic symptoms (N = 2,704, mean age = 41.7 years); although somatic symptoms formed a unique cluster, depression and anxiety symptoms did not.^18^ In a third investigation, the focus of inquiry was the network structure of depression symptoms (N = 3,463, mean age 41 years) corresponding to both *DSM* and non-*DSM* (including anxiety) profiles.^19^ Again, no evidence of clustering based on *DSM* disorders emerged.^19^ Studies of broader psychopathological networks (ie, comprising multiple symptom domains) have also produced inconsistent findings. Boschloo *et al.*^20^ explored the network structure of 120 psychiatric symptoms of 12 *DSM* disorders in the National Epidemiologic Survey on Alcohol and Related Conditions (N = 34,653) and found that depression and anxiety symptoms formed relatively distinct clusters. However, connections between these clusters were common, and clustering may have been affected by the skip logic of the diagnostic interview used. In the only such investigation in adolescents (N = 2,175) to date, depression and anxiety symptoms formed part of a broader internalizing group, rather than unique diagnostic clusters.^21^ These inconsistent findings may be attributable to the use of different measures and samples, and therefore further studies of the network structure of depression and anxiety symptoms are warranted.

Notably, although the above studies were all interested in the clustering of depressive and anxiety symptoms in networks,^17^, ^18^, ^19^, ^20^, ^21^ they relied entirely on visual inspection of network graphs, rather than testing for clustering empirically.^22^, ^23^ Moreover, all but one^21^ of the investigations described above focused on adult samples, and no attention has been paid to changes in symptom networks over development. A developmental investigation will help determine how these symptoms are associated in the early stages of childhood, and whether these associations change as children get older and transition into adolescence. Exploring symptom networks over development will also highlight potential changes in the importance of specific depression and anxiety symptoms as children age. Furthermore, by studying networks longitudinally, it may be possible to gauge the extent to which symptoms feed into and reinforce each other, an often-discussed yet rarely tested facet of the network theory of psychopathology.^13^, ^24^ Thus, the present study uses network analysis to explore the symptom−symptom relationships of parent-reported depression and anxiety from childhood (5 years) through adolescence (14 years), drawing on data from a large longitudinal study. In addressing these research questions, we will contribute to discussions about the classification and development of depression and anxiety. Specifically, this exploratory study seeks to answer three research questions (RQs):

### RQ 1: To what extent are depression and anxiety distinct across development?

The present study will use empirical tests for community structures/modularity as an indicator of the distinctness of the diagnostic boundaries between depression and anxiety over this developmental period. Given the exploratory nature of this study, and the lack of consistency in previous empirical studies of the differentiation of depression and anxiety, no a priori hypotheses regarding the nature/number of clusters are made.

### RQ 2: Do symptom networks become more strongly connected over development?

Changes in overall connectivity will be examined to determine whether the networks as a whole become more interconnected over time, reflecting increased reinforcement among symptoms. If depression and anxiety are found to be distinct constructs (ie, modularity corresponding to diagnostic criteria is observed; RQ1), changes in the strength of cross-domain/bridging edges will be examined to determine whether these constructs become more or less associated over development.

### RQ 3: Does the overall importance of specific depression and anxiety symptoms change over development?

Network centrality will be examined to determine whether certain symptoms become more or less relevant to the overall depression and anxiety networks as children age.

## Method

### Participants

Data were obtained from the National Institute of Child Health and Human Development (NICHD) Study of Early Child Care and Youth Development, a prospective cohort study of children born in 1991 at 10 locations across the United States.^25^ The initial sample comprised 1,364 parent−child pairs. Although the sample was diverse, it was not designed to be nationally representative, in that participating families had higher average income and education and were less likely to be of an ethnic minority.^26^ Ethical approval for the NICHD Study was granted by all data-collecting universities prior to data collection, and at each assessment informed consent was secured from parents and/or teacher. More detailed descriptions of the NICHD Study, including recruitment and assessment procedures, are available elsewhere.^25^ The NICHD Study data are available to researchers (http://www.icpsr.umich.edu/icpsrweb/ICPSR/series/00233).

A total of 12 depression and 6 anxiety symptoms were measured using the *DSM*-oriented scales of the Child Behavior Checklist (CBCL).^6^ The CBCL was completed by study mothers when the children were 5, 6, 8, 9, 10, 11, and 14 years of age. Symptoms are rated on a 3-point scale (0 = not true; 1 = somewhat/sometimes true; 2 = very true/often). Due to low endorsement of severe responses (ie, scores of 2 on individual items), items were rescored to indicate the presence of symptoms (ie, raw responses of 1 or 2 coded as 1) in line with common practice when conducting item-level analyses of the CBCL.^6^, ^27^

### Missing Data

To ensure that attrition did not bias the results, imputation was conducted using the R-package “Amelia,” which implements the expectation-maximization with bootstrapping algorithm.^28^ As network methodologies are currently incompatible with multiply imputed data sets, a single imputed data set based on all participants who provided any CBCL data was produced (N*=* 1,147).

### Statistical Analysis

#### RQ1: To what extent are depression and anxiety distinct across development?

Symptom networks were estimated using the R-package “Isingfit,”^29^ which was developed to construct weighted, undirected networks using binary data. This package employs *elasso*, a methodology based on the Ising^30^ model, in which each variable is regressed on all other variables with an *Ɩ*_1_ (lasso) penalty used to shrink regression coefficients, and set very small coefficients to zero, thus striking a balance between parsimony and explanatory power.^29^ Isingfit produces undirected edges that can be interpreted similarly to partial correlations. Networks were constructed using the 18 symptom nodes at each time point, and were graphically illustrated using the “qgraph” package,^31^ which implements the Fruchterman–Reingold algorithm to place highly connected nodes closer together.^32^ The reliability and accuracy of the estimated networks (ie, the degree of confidence with which edge weight and centrality rankings can be interpreted) were assessed using the “bootnet” package following the guidelines of Epskamp et al.^33^

Modularity was examined to determine whether nodes formed distinct clusters analogous to depression and anxiety, and whether these clusters became more or less distinct over development. A two-step processes was adopted. First, following the guidelines of Dalege *et al.*,^22^ the walktrap algorithm was used to identify community structures within the networks. This algorithm works as follows: (1) starting at a random node, a connection with another node is randomly chosen; (2) this step is repeated multiple times; (3) random walks get “trapped” in densely connected parts of the network (for a more detailed description, see Pons and Latapy^34^). This approach has been shown to effectively identify community structures in psychopathological data.^23^ Next, to quantify the degree of modularity in the networks and to determine whether this increased/decreased over development, the modularity index *Q* was calculated.^35^ This index is calculated by comparing the observed network structure with a randomly connected network.^35^, ^36^ The *Q* index ranges from 0 to 1, with values of >0.3 suggesting that nodes are arranged in nonrandom communities.^35^, ^36^ All modularity analyses were conducted using the R package “igraph.”^37^

#### RQ2: Do symptom networks become more strongly connected over development?

To examine whether the symptom networks became more strongly connected over development, nonparametric permutation tests were employed using the “NetworkComparisonTest” (NCT) package.^24^, ^38^ NCT allows for the comparison of specific edges across networks, and tests invariance in overall connectivity (ie, global strength). This procedure is carried out in three phases. First, the two networks in question are estimated and the relevant test statistics are calculated.^38^ For individual edges, the test statistic is the observed difference in edge weight. For invariance in overall connectivity, the test statistic is the difference in global strength (ie, difference in sum of edge weights of two networks). Second, cases are repeatedly and randomly swapped between networks and the test statistics re-estimated. Third, a reference distribution is created from these test statistics and statistical significance determined, with the *p* value equal to the proportion of test statistics that have a value equal to or greater than the observed test statistic.^38^ Networks were compared using 1,000 random permutations.

#### RQ3: Does the overall importance of specific depression and anxiety symptoms change over development?

To identify the symptoms that were most important to the networks overall (ie, those that had the most frequent/strongest associations with other symptoms), and to examine whether this changed developmentally, three common measures of node centrality were calculated. Strength was calculated by summing the standardized weights of all significant edges in the network.^39^ A node that is high in strength can quickly and directly influence other nodes when activated.^39^ Closeness was calculated by taking the inverse of the sum of the distances of individual nodes from all other nodes in the networks.^40^ High closeness means that a node is likely to be quickly affected by changes in other nodes in the network.^40^ Betweenness was calculated by summing the number of times that each node lay on the shortest path between two other nodes*.* Nodes that are high in betweenness are important for transmitting effects between other nodes in the network. Centrality indices are presented as standardized *z* scores, with higher values indicative of greater importance to the network as a whole.^39^

## Results

### Descriptive Statistics

Item details and frequencies at different ages are presented in Table 1.

**Table 1:** Frequencies of Parent-Reported Symptoms Expressed as a Percentage of the Overall Sample (N= 1,147)

	Age 5	Age 6	Age 8	Age 9	Age 10	Age 11	Age 14
Depression items							
Cries a lot	21	17	15	12	11	8	4
Harms self/attempts suicide	1	1	1	1	1	1	3
Doesn’t eat well	34	27	22	20	20	18	21
Feels worthless	7	10	15	13	13	16	14
Feels too guilty	3	4	6	6	6	6	5
Overtired	15	13	12	12	12	14	20
Sleeps less than most children	13	12	12	12	12	13	11
Sleep more than most children	5	4	4	4	5	5	11
Talks about suicide	2	3	3	2	3	3	2
Trouble sleeping	11	10	12	11	11	10	13
Lacks energy/is underactive	3	5	7	8	11	10	15
Unhappy, sad, or depressed	7	9	13	12	14	13	17
Anxiety items							
Clingy/too dependent	40	28	22	18	20	17	10
Specific fears	39	31	25	22	20	18	11
Fears school	7	5	5	4	4	5	3
Nervous, highstrung, or tense	12	13	18	19	19	19	18
Too fearful or anxious	10	12	12	10	12	11	9
Worries	23	27	34	32	36	33	28

### Research Questions

#### RQ1: To what extent are depression and anxiety distinct across development?

**Figure 1:**
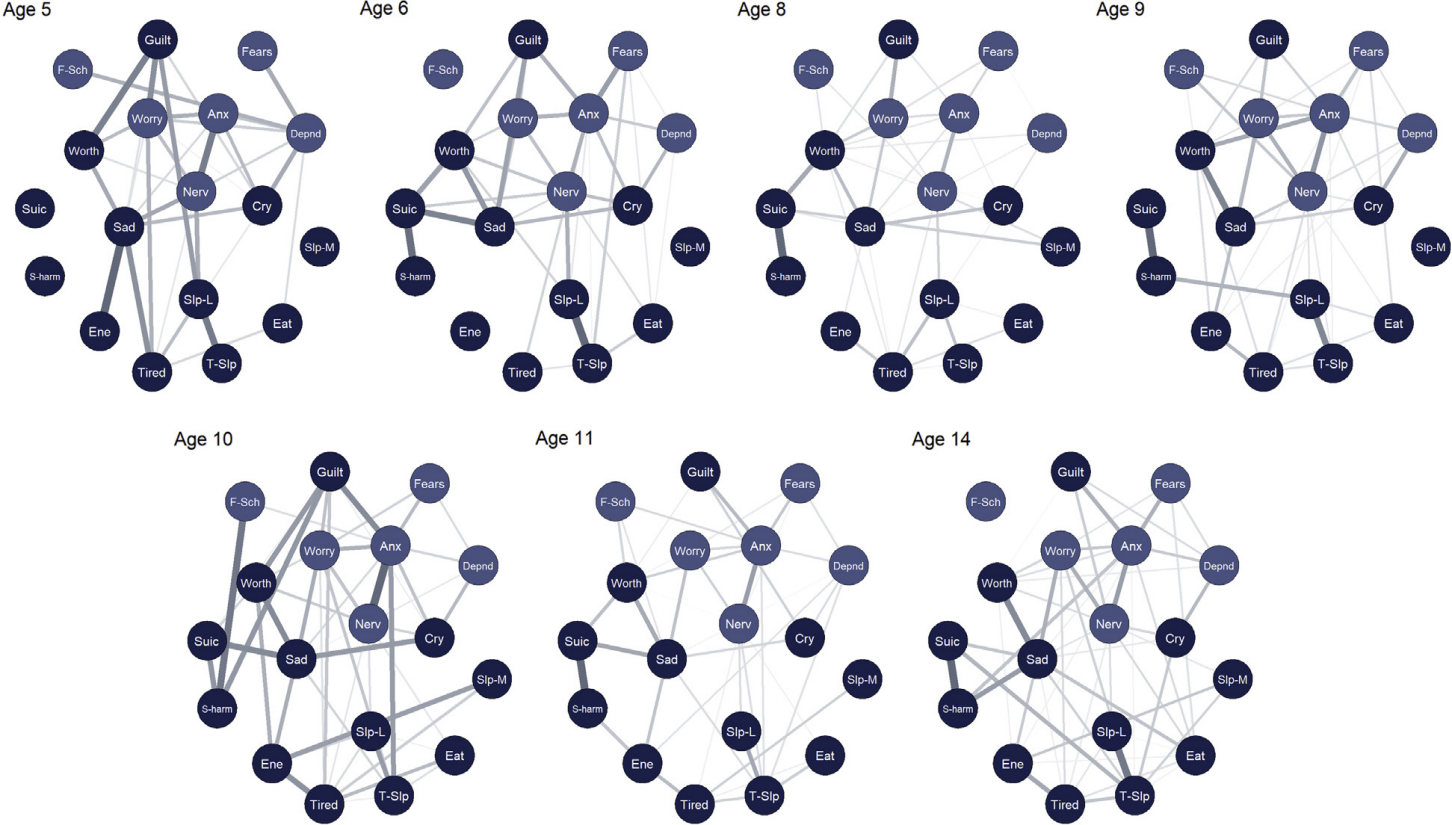
Symptom Networks: *DSM* Oriented Depression and Anxiety Scales (N= 1,147) ***Note:****All edges positive. Node placement reflects average layout over time. Node coloring reflects scoring of DSM-oriented scales. Anx = too fearful or anxious; cry = cries a lot; depnd = cling/too dependent; eat = doesn't eat well; ene = lacks energy/underactive; f-sch = fears school; fears = specific fears; guilt = feels too guilty; nerv = nervous, high strung, or tense; s-harm = harms self/attempt suicide; sad = uphappy, sad, or depressed; slp-L = sleeps less than most children; slp-M = sleeps more than most children; suic = talks about suicide; t-slp = trouble sleeping; tired = overtired; worry = worries; worth = feels worthless. Please note color figures are available online.*

Networks were constructed separately at each time point and are presented in Figure 1 (color coded corresponding to *DSM*-oriented scales). For ease of visual comparison, the networks were restricted to a consistent “average layout,” presented across all ages.

For networks presented with their unique layouts, see Figures S1 to S7, available online. Notably, symptoms of depression and anxiety did not cluster into distinct regions of space; symptoms were highly interconnected within and across the two domains. Of a possible 153 edges, between 100 (65%; age 14 years) and 120 (78%; age 5 years) were above zero. Over time, there was variation in the edges that were strongest (see Table S1, available online). The two most consistently strong edges were “Talks about suicide – Harms self” and “Sleeps less – Trouble sleeping.” In the case of “Sleeps less – Trouble sleeping,” this was likely due to the similarity of the items. The edges “Nervous – Anxious” and “Sad – Worthless” were also consistently among the strongest edges. Of the 10 strongest edges at each time point, many linked depression and anxiety symptoms. “Guilt” played a large role in linking both domains, demonstrating frequent strong edges with “Anxious” and “Worries.” Other common bridging edges were “Worries – Sad” and “Cries – Too dependent.”

**Figure 2:**
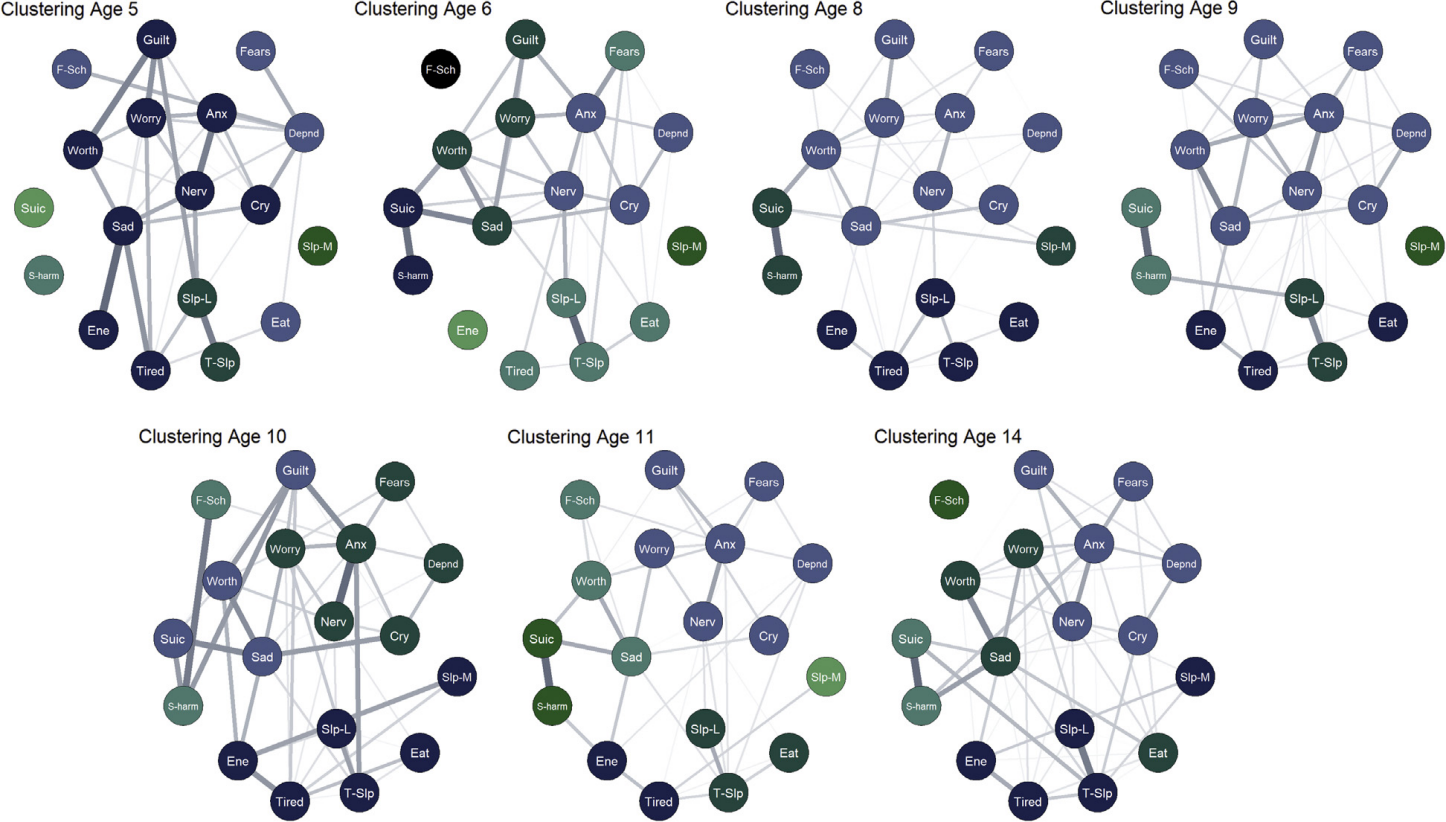
Symptom Networks: Walktrap Clustering (N = 1,147) ***Note:****All edges positive. Node coloring reflects clusters identified using walktrap algorithm. Anx = too fearful or anxious; cry = cries a lot; depnd = cling/too dependent; eat = doesn't eat well; ene = lacks energy/underactive; f-sch = fears school; fears = specific fears; guilt = feels too guilty; nerv = nervous, high strung, or tense; s-harm = harms self/attempt suicide; sad = uphappy, sad, or depressed; slp-L = sleeps less than most children; slp-M = sleeps more than most children; suic = talks about suicide; t-slp = trouble sleeping; worry = worries; worth = feels worthless. Please note color figures are available online.*

The results from the modularity analyses (indicating the extent to which nodes formed distinct clusters) are presented graphically in Figure 2.

**Figure 3:**
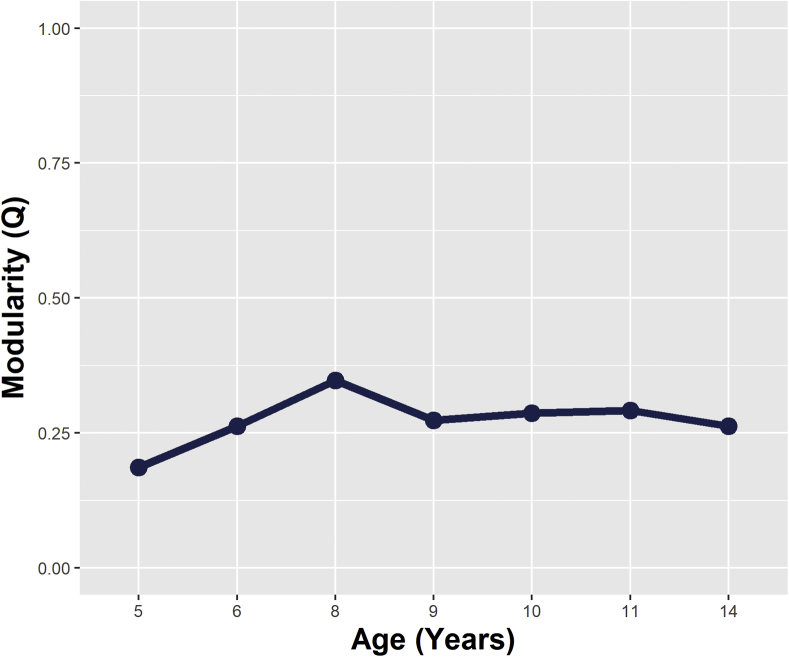
Network Modularity Values (*Q*-Index) Over Time ***Note:****Values <0.3 indicate that clusters are random.*

There was no evidence of clustering in the form of distinct depression and anxiety constructs. Aside from age 8 years, all *Q* values were less than 0.3 (Figure 3), indicating that any clustering identified within these networks was likely random. Again, this demonstrates that depression and anxiety items did not form distinct clusters over this developmental period.

For the results of the bootstrapped difference tests, see Figures S8 to S14, available online. There were few significant differences between the strongest edges; as such, the ranking of edge weights should be interpreted with some caution. The correlation stability (CS) coefficients were generally low, ranging from 0.05 to 0.36, indicating that the rank ordering of the centrality indices should also be interpreted with a degree of care (see Figures S15−S21, available online).

#### RQ 2: Do symptom networks become more strongly connected over development?

**Figure 4:**
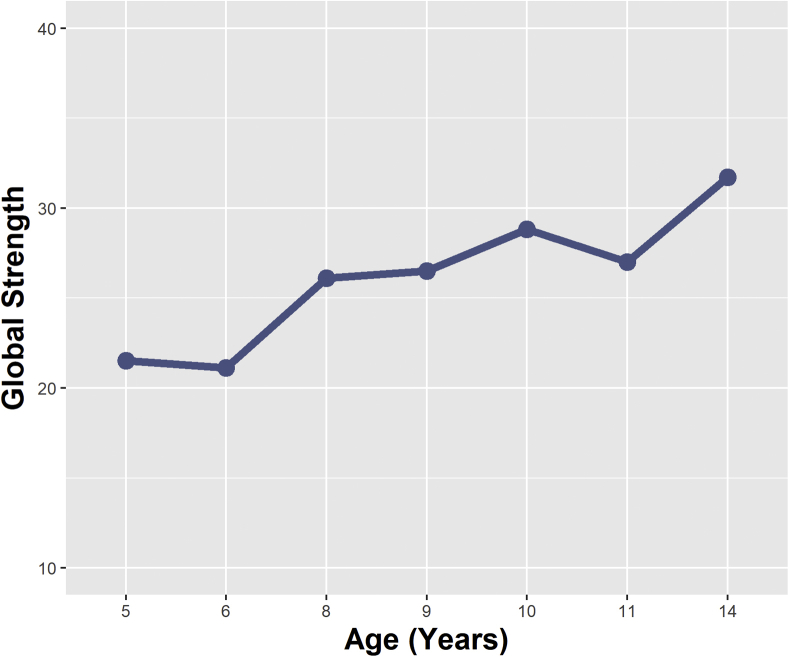
Global Strength Values Over Time (Indicating the Overall Connectivity Within the Network)

Given the lack of evidence of distinct clustering corresponding to depression and anxiety disorder domains, changes in overall connectivity (rather than specific cross-domain paths) were explored using NCT permutation tests. Global strength values are presented in Figure 4. Aside from minor declines at age 6 and 11 years, global strength appeared to increase in a linear fashion over time. Year-on-year there was no significant differences in global strength; however, there were significant differences between values in early childhood and adolescence (Δglobal strength = 10.20, *p* = .028 [5 versus 14 years], Δglobal strength = 10.59, *p* = .036 [6 versus 14 years]).

#### RQ3: Does the overall importance of specific depression and anxiety symptoms change over development?

**Figure 5:**
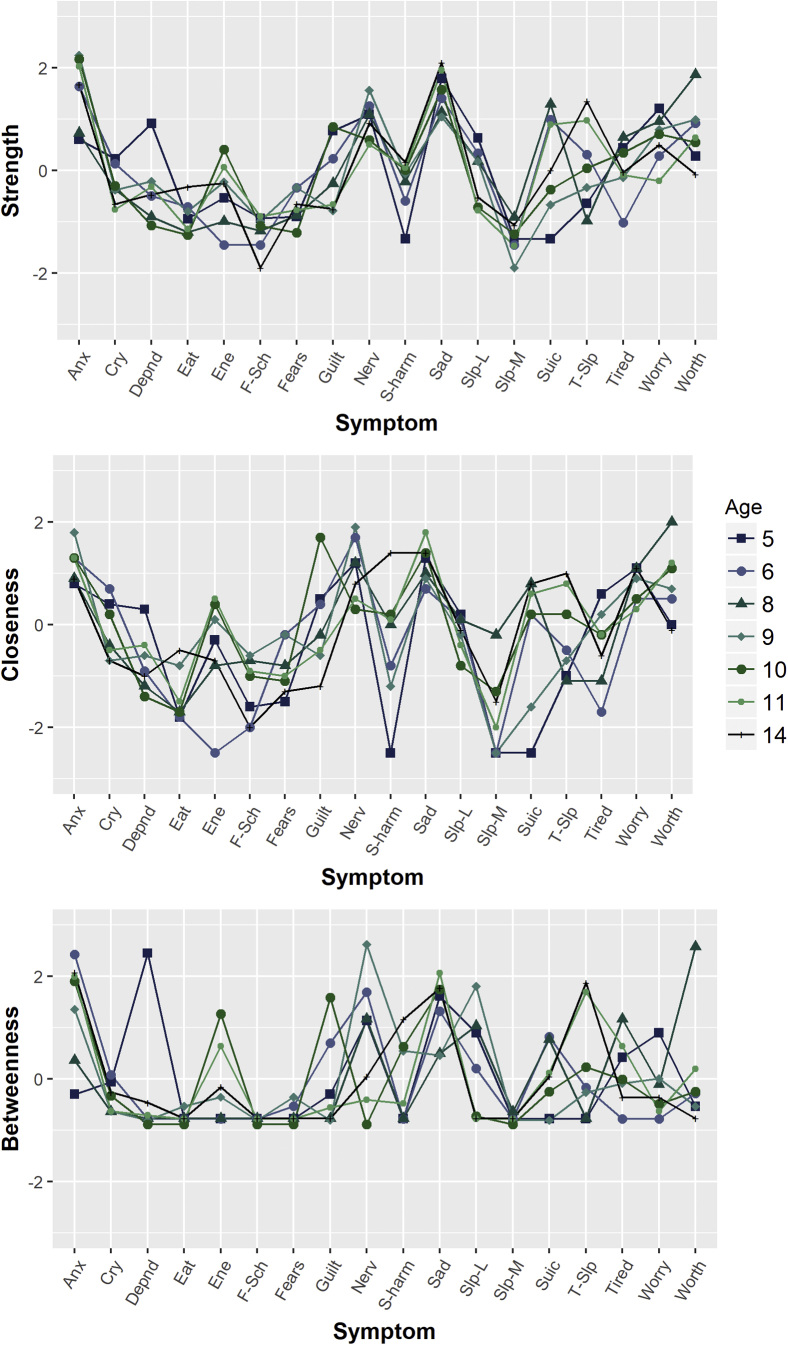
Centrality Indices Demonstrating Importance of Each Symptom Within the Network Over Time ***Note:****Centrality values presented as standardized scores on y-axis. Anx = too fearful or anxious; cry = cries a lot; depnd = cling/too dependent; ene = lacks energy/underactive; f-sch = fears school; nerv = nervous, high strung, or tense; s-harm = harms self/attempt suicide; slp-L = sleeps less than most children; slp-M = sleeps more than most children; suic = suicide; t-slp = trouble sleeping. Please note color figures are available online.*

Centrality indices (which indicate overall importance of a given node to the network) are presented in Figure 5. Overall, the most central nodes were “Anxious,” “Sad,” “Nervous,” and “Worthless.” The rank orderings of centrality indices were reasonably consistent over time for the anxiety items; “Anxious” generally had the highest strength and betweenness values, followed by “Nervous” and “Worries.”

There was greater developmental variation in the centrality of depression items; “Sad” consistently had the highest strength and betweenness across time, whereas the centrality of other depression items varied considerably. “Sleeps more than other children” and “Fears school” were consistently the least central items.

## Discussion

This exploratory study is the first to examine the network structure of depression and anxiety symptoms from a developmental perspective in a cohort of children as they progress from early childhood to mid-adolescence. We found a highly interconnected symptom network, with no evidence of modularity corresponding to the domains of depression and anxiety. There was some evidence of an increase in overall connectivity through development, and although there was variation in the importance of symptoms over time, the most central and least central symptoms in the network were generally consistent.

The lack of distinct clustering and frequent and strong bridging edges suggest that there is little to demarcate depression and anxiety symptoms from childhood through adolescence. Although the present study used data from a general population cohort, similar findings have emerged in clinical samples of adults.^18^, ^19^ Thus, the current study indicates that this is the case from at least early childhood. These findings challenge the view that depression and anxiety represent wholly distinct constructs during this developmental period: rather it appears that both domains of symptomatology form part of a larger psychopathological network, wherein individual symptoms are associated both directly and indirectly through other symptoms in the network.^19^ This highlights the complexity of the structure of these common forms of psychopathology. As such, when an investigation focuses on depression or anxiety in isolation, important information likely is lost. This may go some way to explain why etiological research has struggled to identify consistent unique risk factors, both biological and environmental, for depression and anxiety.^41^, ^42^

Cross-domain edges were frequent and strong, and most often involved the symptoms “Guilt,” “Worries,” and “Cries.” These symptoms may be considered bridging symptoms and go some way to explaining the correlations when depression and anxiety are treated as distinct constructs, at least in a statistical sense. Whether these associations between symptoms represent causal pathways is up for debate. Take, for example, the common edge “Worries – Sad.” It is plausible that sustained arousal due to worry could lead the body to experience emotional and physiological exhaustion, culminating in feelings of low mood.^3^ This low mood may then feed into other depressive symptoms, such as feelings of worthlessness. In such a case, the edge “Worries – Sad” would reflect a substantive mechanistic process. Alternatively, it could be that certain “bridge symptoms” are common outcomes of two unrelated nodes, for example, “Too dependent” → “Cries” ← “Sad.” That a bridge edge might represent an unmeasured common cause (e.g., genetic vulnerability, environmental risk, emotional development mechanisms) represents a third potential explanation.^16^ These possibilities highlight the complex and varying ways by which individual symptoms may be associated, and in turn give rise to correlation at the construct level.

Indeed, shared etiological and mechanistic explanations for edges are not necessarily mutually exclusive; external etiological agents may influence one or more symptom nodes, which in turn spread their effects through other symptom nodes, eventually settling into a state of mutual reinforcement.^16^, ^43^ If symptoms are feeding into and reinforcing each other in this manner, one would expect to see the associations between items become stronger over time.^44^ In the present study, there was some evidence of a linear increase in connectivity over time. Although global strength values did not significantly increase year-on-year, connectedness was significantly higher at age 14 compared with ages 5 and 6. This suggests that mutual reinforcement may occur throughout development, leading to children with higher levels of symptoms in early childhood being more vulnerable to symptoms in later years, a finding observed in longitudinal studies focusing on construct-level depression and anxiety.^45^ Further research using developing network methodologies may help quantify this reinforcement and further unpack the mechanisms by which depression and anxiety symptoms are related, and thus help identify key targets for intervention. For example, longitudinal networks based on experience sampling data show promise in this regard.^46^

With regard to the overall importance of symptoms, the most central symptoms were generally stable over this developmental period. Core symptom nodes included “Anxious,” “Sad,” “Nervous,” and “Worthless.” This suggests that these symptoms may be key factors in the onset/maintenance of networks of mixed depressive and anxiety symptoms across development. These items appear to represent quite global negative affect states, which may be thought of as most closely mirroring the underlying neurobiological systems subserving negative valence^47^ or the core appraisals within a cognitive−behavioral framework linked to perceived threat or loss. Their centrality is thus quite consistent with a number of transdiagnostic approaches to research and clinical practice. Other nodes demonstrated developmental variation in their overall importance. For instance, “Clingy/too dependent” demonstrated higher centrality at the earliest assessment (age 5 years), before reducing and leveling off at later time points, suggesting that this may be a more important indicator of anxiety/depression at younger ages. This pattern mirrors developmental profiles of internalizing symptoms in early childhood.^48^

The main strength of the present study was the longitudinal design, with anxiety and depression symptoms assessed at several time points across development, which allowed us to explore developmental changes in the relationships between these symptom networks. With regard to limitations, the networks demonstrated relatively low accuracy and reliability, as assessed using bootstrapped difference tests. There are two explanations for this, the first of which is that there are generally only minor differences in the strengths of edges both within and across depression and anxiety symptoms. This would further support the lack of distinct boundaries between depression and anxiety during this developmental phase. Alternatively, the conservative nature of this statistical test may be obscuring actual differences, as Epskamp *et al.*^33^ note that at typical sample sizes used in psychological research, it will likely identify fewer significant differences than exist in the population. The present study also relied solely on maternal reports, and it is worth noting that when children become old enough to self-report, cross-informant agreement has generally been low.^49^ Finally, it must be acknowledged that the network approach to psychopathology remains in its infancy, and debates continue regarding the most appropriate network methodologies.^50^, ^51^, ^52^, ^53^ The methods used in the present study, as with previous studies of depression and anxiety networks, were exploratory in nature; therefore, the development of means to test confirmatory hypotheses in a network framework will allow us to better examine the replicability of findings across studies.^54^ This is important, given that certain aspects of different measures (e.g., content, symptoms covered, skip-patterns) may have an impact on the network structure of a given construct.^20^

In conclusion, the findings from this exploratory study highlight the strong interconnectivity of depression and anxiety symptoms from a very early age at the general population level. As such, they lend support to arguments to reconsider the way in which these disorders are classified. We find that the manner in which symptoms are related and their relative importance within the network remains fairly consistent over this wide developmental period, thus providing useful evidence about the structure of these disorders through development. Increases in connectivity suggest that symptoms might reinforce each other through development, helping to explain the high levels of lifetime continuity of and between these disorders.
