# Supplementary material for: Networks of Depression and Anxiety Symptoms Across Development
Source: J Am Acad Child Adolesc Psychiatry. 2018 Dec;57(12):964–73. doi: 10.1016/j.jaac.2018.05.027 (PMC6290121; doi:10.1016/j.jaac.2018.05.027)
Supplement: Table S1 and Figures S1-S21 [file mmc1.docx]

| **Table S1. Strongest edges at each age** | | | | | | |
| --- | --- | --- | --- | --- | --- | --- |
| **Age 5** | **Age 6** | **Age 8** | **Age 9** | **Age 10** | **Age 11** | **Age 14** |
| Un−act−−Sad | Slp−L−−T−Slp | S−harm−−Suic | S−harm−−Suic | Nerv−−Anx | S−harm−−Suic | S−harm−−Suic |
| Slp−L−−T−Slp | S−harm−−Suic | Wrth−−Suic | Wrth−−Sad | S−harm−−F−Sch | Nerv−−Anx | Slp−L−−T−Slp |
| Nerv−−Anx | Suic−−Sad | Guilt−−Worry | Slp−L−−T−Slp | Suic−−Sad | Suic−−Sad | Wrth−−Sad |
| Wrth−−Guilt | Wrth−−Sad | Nerv−−Anx | Nerv−−Anx | Wrth−−Sad | Slp−L−−T−Slp | S−harm−−Sad |
| Guilt−−Worry | Fears−−Anx | Wrth−−Sad | Wrth−−Anx | Guilt−−Anx | Wrth−−Sad | Nerv−−Anx |
| Tired−−Sad | Wrth−−Suic | Tired−−Slp−L | Cry−−Depnd | Tired−−Un−act | Guilt−−Anx | Tired−−Un−act |
| Guilt−−Slp−L | Guilt−−Sad | Slp−L−−T−Slp | Nerv−−Worry | S−harm−−Suic | Tired−−Un−act | Nerv−−Worry |
| Sad−−Nerv | Slp−L−−Nerv | Cry−−Sad | S−harm−−Slp−L | S−harm−−Guilt | Wrth−−Suic | Sad−−Worry |
| Wrth−−Sad | Guilt−−Anx | Sad−−Worry | Sad−−Worry | Cry−−Sad | Sad−−Worry | Fears−−Anx |
| Cry−−Depnd | Nerv−−Anx | Wrth−−Worry | Tired−−Un−act | Wrth−−Guilt | Un−act−−Sad | Guilt−−Anx |
| **Key** |  |  |  |  |  |  |
| Depression | Anxiety | Cross-Domain |  |  |  |  |


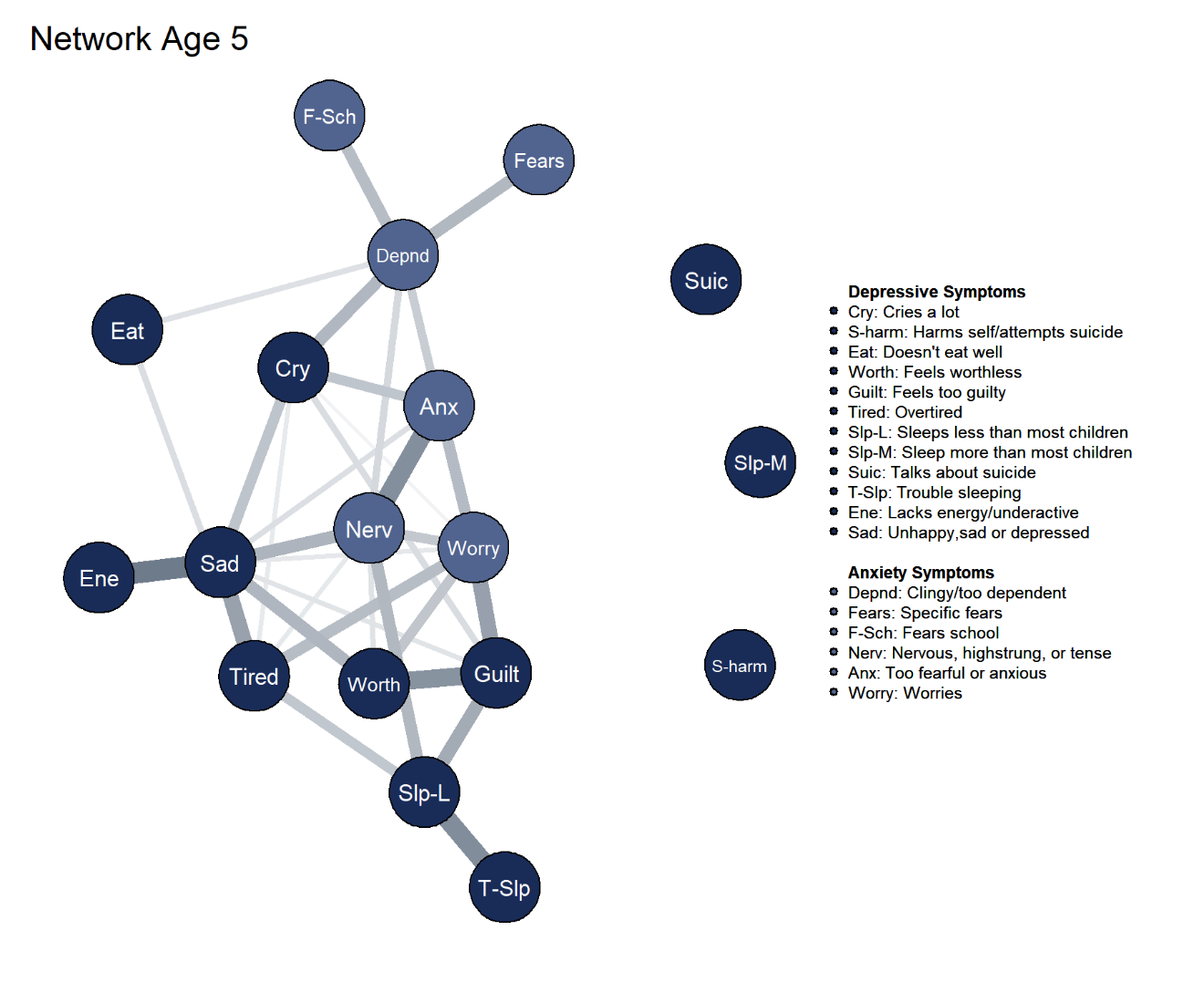


Figure S1. Association network (unique layout) at age 5.


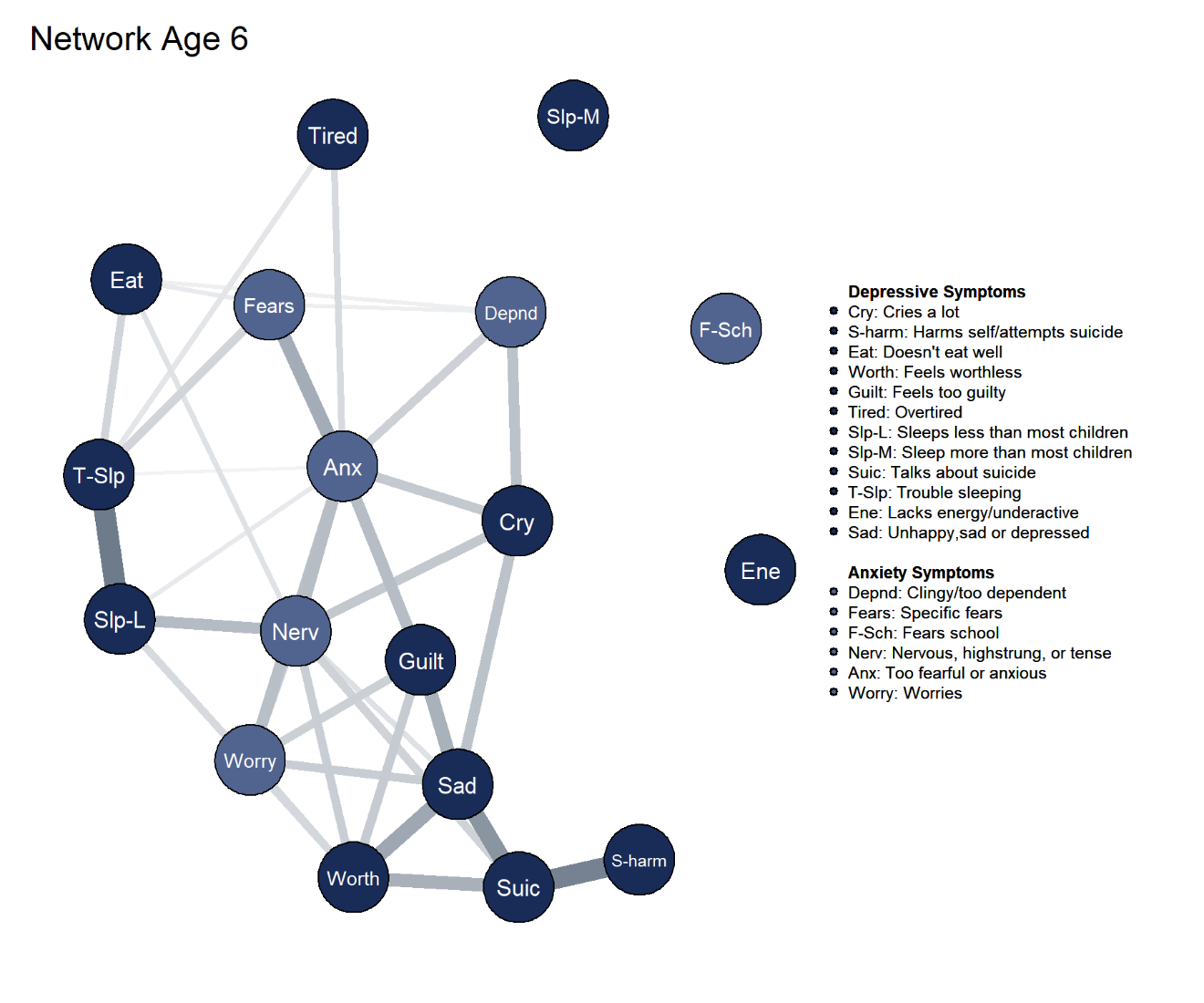


Figure S2. Association network (unique layout) at age 6.


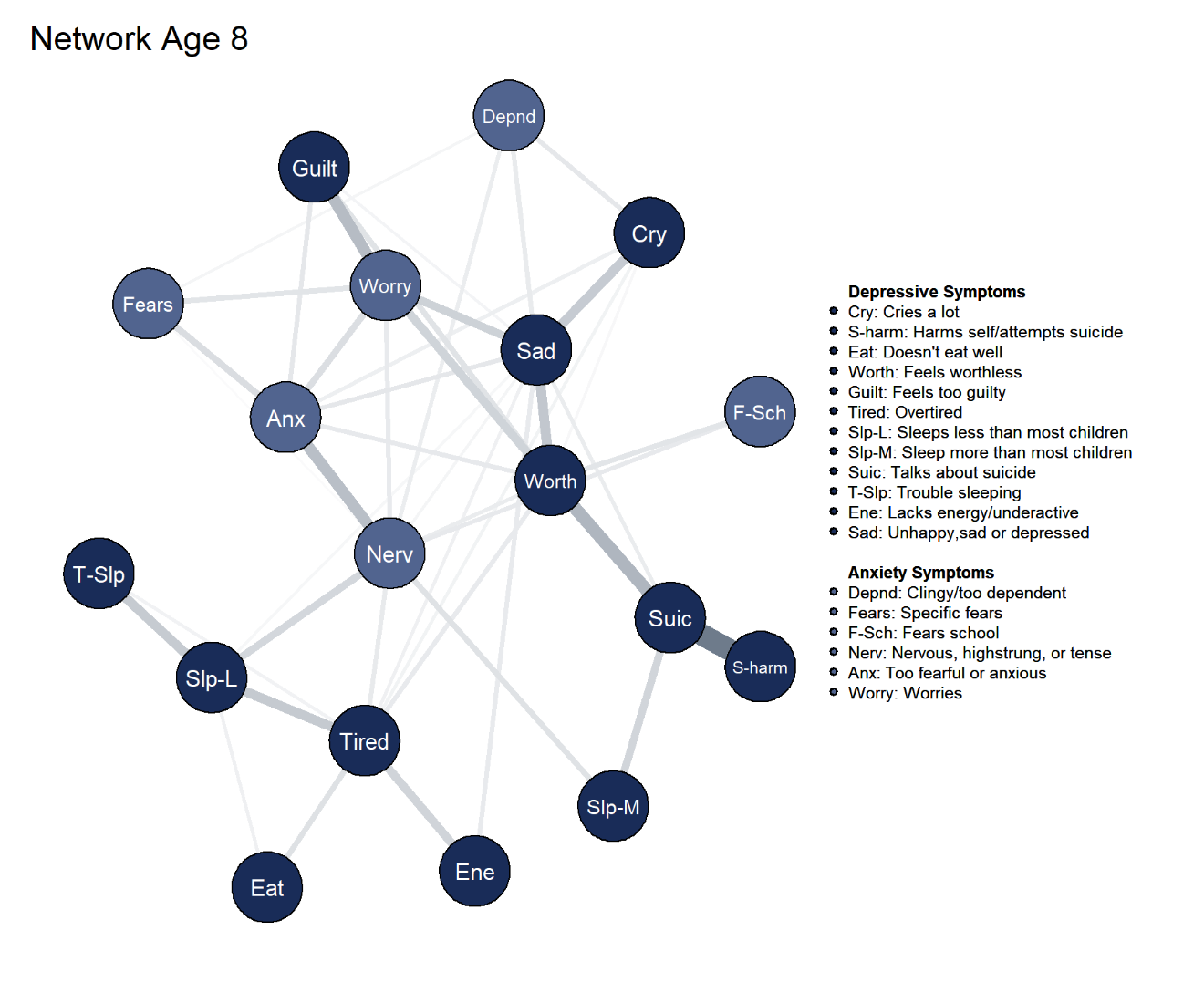


Figure S3. Association network (unique layout) at age 8.


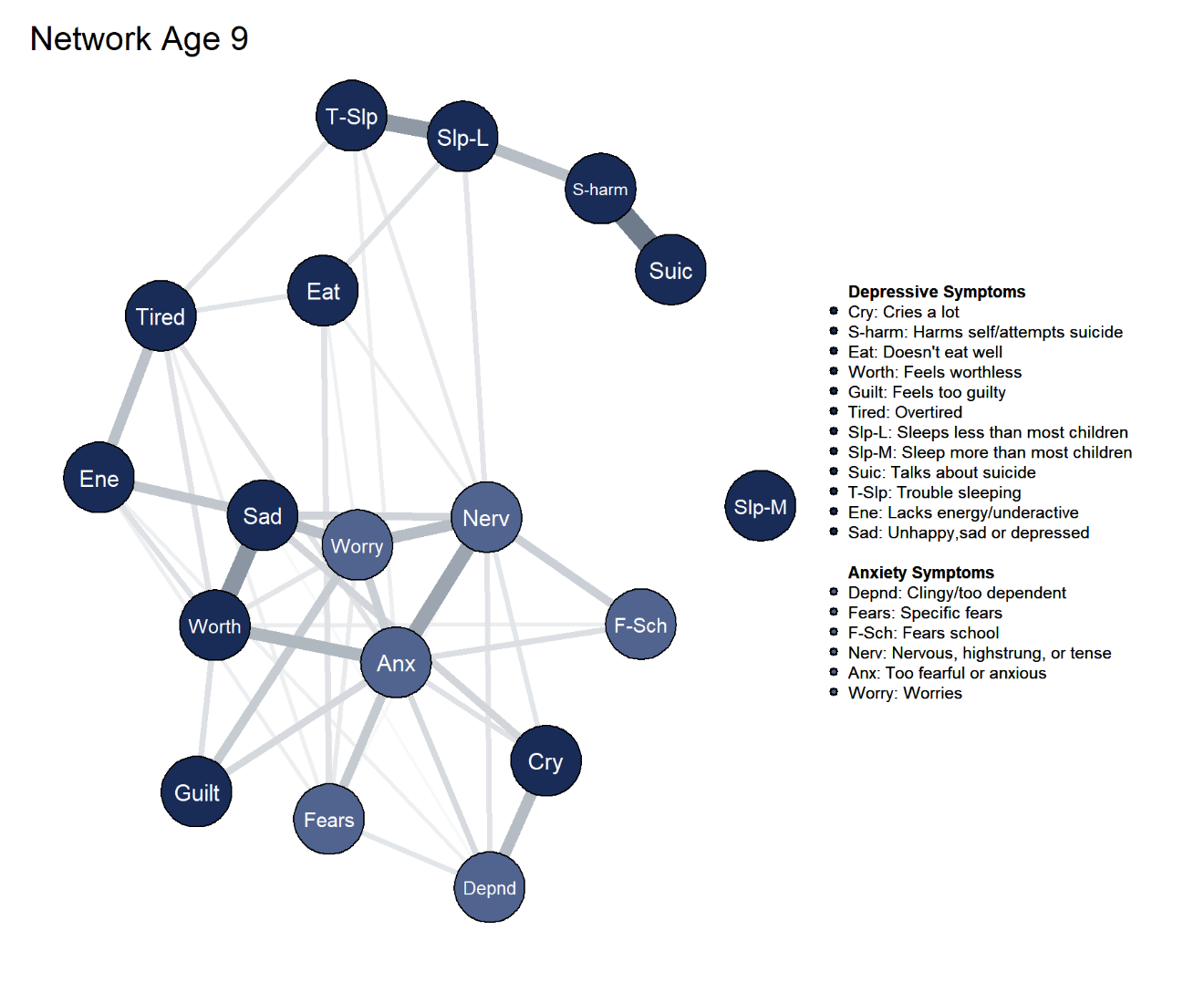


Figure S4. Association network (unique layout) at age 9.


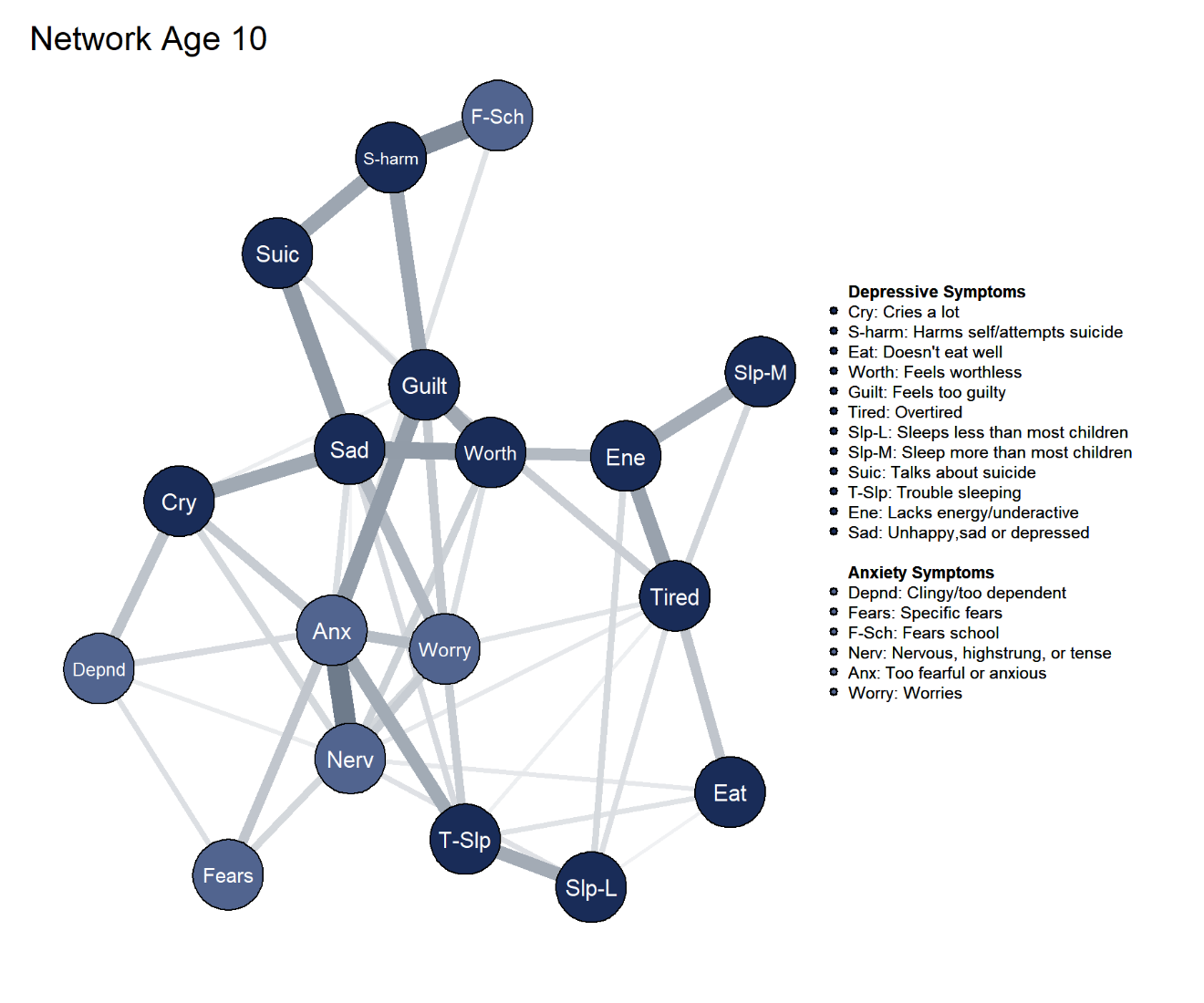


Figure S5. Association network (unique layout) at age 10


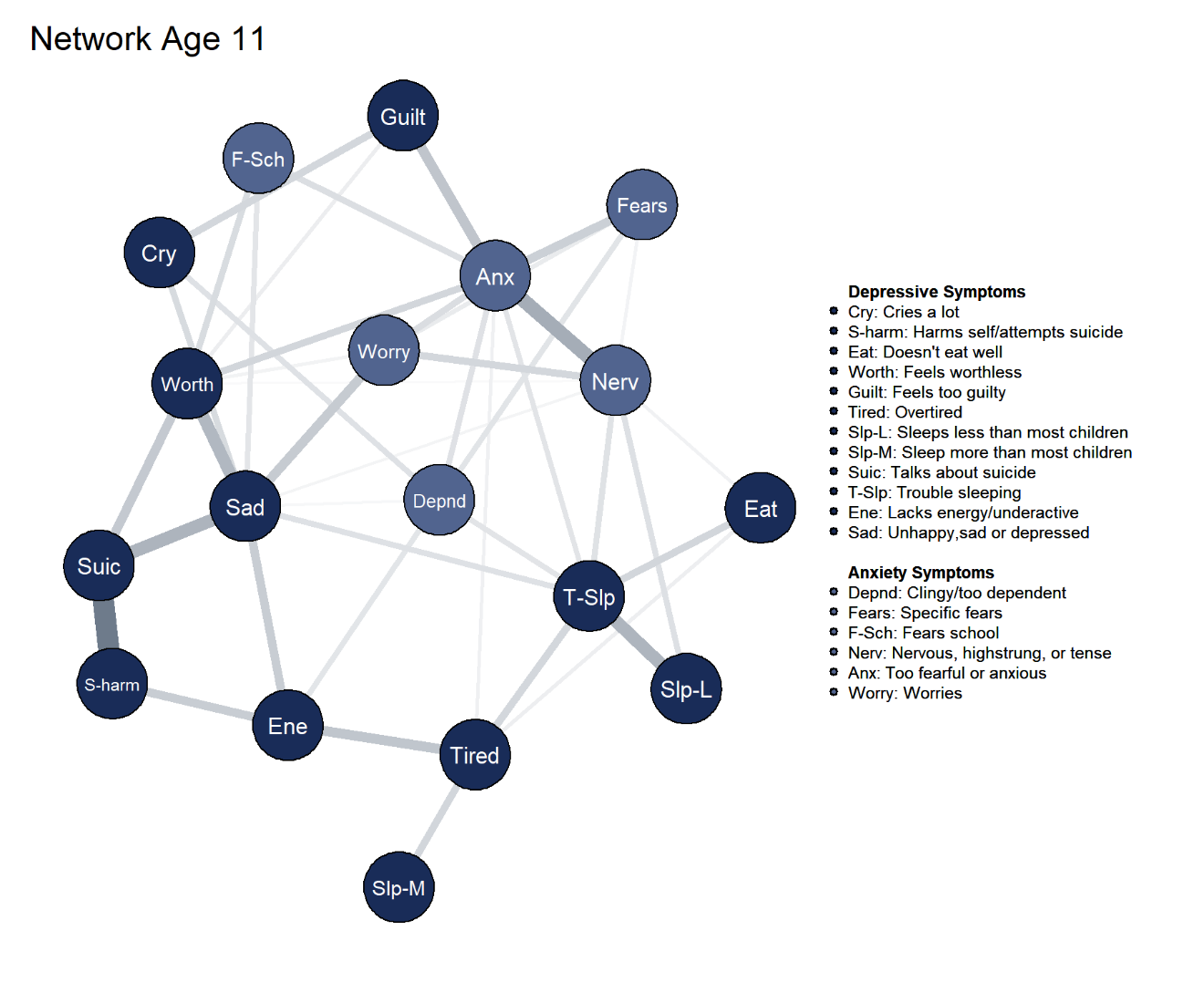


Figure S6. Association network (unique layout) at age 11.


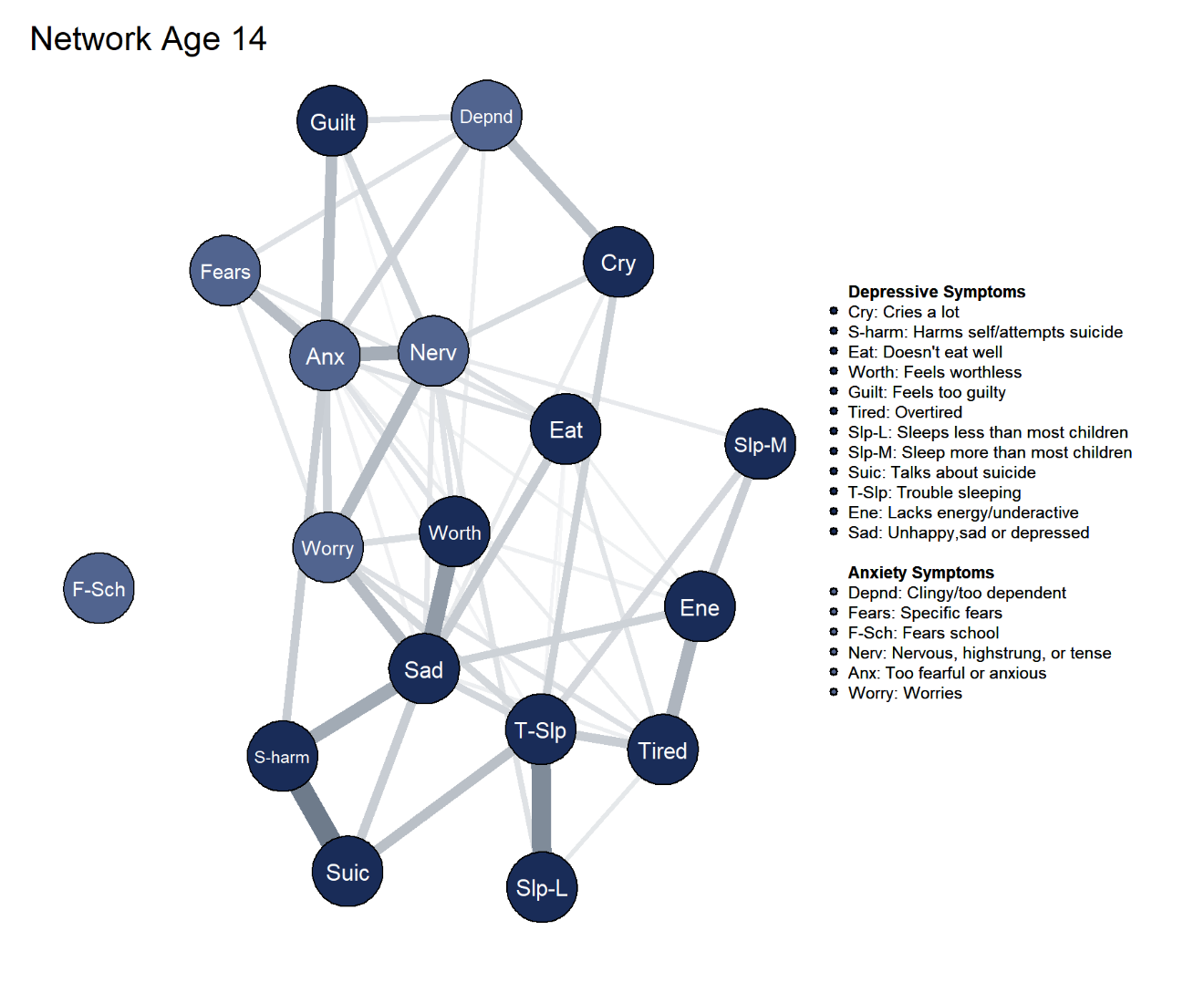


Figure S7. Association network (unique layout) age 14.

Figure S8. Bootstrapped difference tests between non-zero edges at age 5 years. Black squares indicate significant differences between edges (ꭤ = (05), whereas grey boxes indicate no significant difference.

Figure S9. Bootstrapped difference tests between non-zero edges at age 6 years. Black squares indicate significant differences between edges (ꭤ = (05), whereas grey boxes indicate no significant difference.

Figure S10. Bootstrapped difference tests between non-zero edges at age 8 years. Black squares indicate significant differences between edges (ꭤ = (05), whereas grey boxes indicate no significant difference.

Figure S11. Bootstrapped difference tests between non-zero edges at age 9 years. Black squares indicate significant differences between edges (ꭤ = (05), whereas grey boxes indicate no significant difference.

Figure S12. Bootstrapped difference tests between non-zero edges at age 10 years. Black squares indicate significant differences between edges (ꭤ = (05), whereas grey boxes indicate no significant difference.

Figure S13. Bootstrapped difference tests between non-zero edges at age 11 years. Black squares indicate significant differences between edges (ꭤ = (05), whereas grey boxes indicate no significant difference.

Figure S14. Bootstrapped difference tests between non-zero edges at age 14 years. Black squares indicate significant differences between edges (ꭤ = (05), whereas grey boxes indicate no significant difference.

Figure S15. Mean correlations between centrality values of original sample (age 5) and sub samples with different degrees of persons dropped. Lines reflect means and areas around the lines reflect 95% CIs.

Figure S16. Mean correlations between centrality values of original sample (age 6) and sub samples with different degrees of persons dropped. Lines reflect means and areas around the lines reflect 95% CIs.

Figure S17. Mean correlations between centrality values of original sample (age 8) and sub samples with different degrees of persons dropped. Lines reflect means and areas around the lines reflect 95% CIs.

Figure S18. Mean correlations between centrality values of original sample (age 9) and sub samples with different degrees of persons dropped. Lines reflect means and areas around the lines reflect 95% CIs.

Figure S19. Mean correlations between centrality values of original sample (age 10) and sub samples with different degrees of persons dropped. Lines reflect means and areas around the lines reflect 95% CIs.

Figure S20. Mean correlations between centrality values of original sample (age 11) and sub samples with different degrees of persons dropped. Lines reflect means and areas around the lines reflect 95% CIs.

Figure S21. Mean Correlations Between Centrality Values of Original Sample (Age 14) and Sub-Samples With Different Degrees of Persons Dropped. Lines Reflect Means And Areas Around The Lines Reflect 95% Cis.
